# An Effective Approach for the Multiobjective Regional Low-Carbon Location-Routing Problem

**DOI:** 10.3390/ijerph16112064

**Published:** 2019-06-11

**Authors:** Longlong Leng, Yanwei Zhao, Jingling Zhang, Chunmiao Zhang

**Affiliations:** Key Laboratory of Special Equipment Manufacturing and Advanced Processing Technology, Ministry of Education, Zhejiang University of Technology, Hangzhou 310023, China; cyxlll@zjut.edu.cn (L.L.); jlzhang@zjut.edu.cn (J.Z.); zhcm@zjut.edu.cn (C.Z.)

**Keywords:** regional low-carbon location-routing problem, multiobjective optimization, fuel consumption, carbon emission, multiobjective hyperheuristics

## Abstract

In this paper, we consider a variant of the location-routing problem (LRP), namely the the multiobjective regional low-carbon LRP (MORLCLRP). The MORLCLRP seeks to minimize service duration, client waiting time, and total costs, which includes carbon emission costs and total depot, vehicle, and travelling costs with respect to fuel consumption, and considers three practical constraints: simultaneous pickup and delivery, heterogeneous fleet, and hard time windows. We formulated a multiobjective mixed integer programming formulations for the problem under study. Due to the complexity of the proposed problem, a general framework, named the multiobjective hyper-heuristic approach (MOHH), was applied for obtaining Pareto-optimal solutions. Aiming at improving the performance of the proposed approach, four selection strategies and three acceptance criteria were developed as the high-level heuristic (HLH), and three multiobjective evolutionary algorithms (MOEAs) were designed as the low-level heuristics (LLHs). The performance of the proposed approach was tested for a set of different instances and comparative analyses were also conducted against eight domain-tailored MOEAs. The results showed that the proposed algorithm produced a high-quality Pareto set for most instances. Additionally, extensive analyses were also carried out to empirically assess the effects of domain-specific parameters (i.e., fleet composition, client and depot distribution, and zones area) on key performance indicators (i.e., hypervolume, inverted generated distance, and ratio of nondominated individuals). Several management insights are provided by analyzing the Pareto solutions.

## 1. Introduction

City logistics poses challenges to governments, businesses, carries, and citizens, particularly in the context of freight transport [1] in terms of three effects: economy, society, and the environment. Therefore, it requires new business operating models for addressing the above triple effects. Meanwhile, with increasing public demand for sustainable development and health, city logistics, as a primary source of carbon emissions (CE), should initiate reductions in CE during related activities. In the last few decades, many researchers have developed tools to optimize logistics networks, especially the location-routing problem (LRP), which includes location-allocation and vehicle-routing problems (VRP) [2]. However, the basic LRP model is only concerned with logistics costs and neglects the environmental and social effects of transportation.

Recently, the permutation and coordination of economic, social, and environmental benefits have emerged as one of the most addressed problem. The low-carbon LRP (LCLRP) concept introduced by Zhang et al. [3] focuses on depot locations and vehicle routes to minimize total CE from depots and traveling activities. The regional LCLRP (RLCLRP), which was first proposed by Koc et al. [1], considers the distribution of clients and depots located in nested zones characterized by different speed limits. Leng et al. [2] further developed a single-objective model (SOM) for the RLCLRP, with consideration of a set of given real-world constraints. The above three papers all share the same feature, namely SOMs. In real-life, however, multiobjective problems (MOP) always play a role in determining trade-offs among multiple demands. Therefore, Leng et al. [4] developed a biobjective model for the RLCLRP and quantum-inspired selection to improve hyper-heuristic performance. Meanwhile, for the other variants of LRP, there were several studies devoted to the development of economic and environmental effects. For example, Pourhejazy et al. [5] considered the fuel consumption in a variant of LRP, and modeled a biobjective model for their problem.

With the development of economy and society, joint effects of logistics on economy, society, and environment should be considered simultaneously rather than separately. As the main performance indicators of logistics network, our proposed model is in tune with the above triple concerns. In other words, the first objective defined FCCE as a traveling cost (economic and environmental effect), delivery duration (i.e., total traveling time) as the second objective, and client satisfaction (social effect) (i.e., total client waiting time) as the third objective.

In real world, most logistics enterprises don’t consider and analyze the joint effects in city logistics as they are only pursuing economic profit. Additionally, our study follows the basic idea of Leng et al. [4] and defines a multiobjective model (MOM) for the RLCLRP (MORLCLRP). To date, no research has been conducted using MOMs as a means to minimize logistics cost, service duration, and client waiting time, simultaneously. Furthermore, fewer studies have incorporated fuel consumption and carbon emission (FCCE) into basic LRP models, such as Pourhejazy et al. [5] and see Section 2.3. Indeed, most studies have failed to handle FCCE appropriately, in other words, the FCCE should be used as the traveling cost like [1,2,3] instead of an objective or constraints (see Section 2.2). The main contributions are as follows:

1. We introduced a mixed-integer linear programming MOM for the RLCLRP. In the real-world logistics network, cost, service duration, and client satisfaction were considered the most significant performance indicators, and traveling cost incorporated FCCE cost.
2. We developed an efficient MOHH to solve the MORLCLRP. For HLHs, we provided four selection strategies and three acceptance criteria to improve the performance of the MOHH framework and developed three MOEAs as the pool of LLHs.
3. We conducted extensive computational experiments to assess the efficiency of the proposed algorithms and developed managerial implications by assessing problem parameters, such as client and depot locations, speed zone areas, and fleet composition. The model, algorithms and computational results can serve as a stepping-stone for further MORLCLRP research with cold chain logistics [6].

The paper is structured as follows: Section 2 provides a review of related literature; Section 3 describes the MORLCLRP with simultaneous pickup and delivery, heterogeneous fleet, and hard time windows; Section 4 gives a brief description of the hyper-heuristic framework together with a general MOEA structure for the MORLCLRP; Section 5 describes the computational experiments and simulated results; and, Section 6 outlines the study conclusions.

## 2. Literature Review

An important objective of the current research was to acquire a set of Pareto solutions for the MORLCLRP within the logistics network. We reviewed related literature based on models estimating FCCE, factors affecting FCCE, and the LCLRP and RLCLRP. As to the literature about the solution methods for the LRP and its variants, the reader is referred to the surveys of Pourhejazy et al. [7] and Drexl and Schneider [8].

### 2.1. Research Considering Effecting Factors

A good estimation model requires analysis of influencing factors. Ardekani et al. [9], Bigazzi and Bertini [10], Alwakiel [11], Lin et al. [12] and Demir et al. [13,14] investigated the factors influencing FCCE, with five categories being identified as a result: (1) vehicle-specific, including rough mass, shape, engine size/type, and fuel type; (2) environmental, including roadway gradient, wind, ambient temperature, altitude, pavement type, and surface conditions; (3) traffic conditions, including congestion, speed limit, and traffic signal; (4) driver behavior, including operation level (e.g., shifting skill) and idle control; and (5) operational-related including fleet size and composition, load, and number of stops. Among the above factors, vehicle speed and vehicle-specific parameters are the most significant.

As speed is affected by inertia, air/rolling resistance, and road slope, it is one of the most important factors affecting FCCE [14]. Koc et al. [1] determined the optimal speed that minimizes FCCE. Demir et al. [15] also derived an optimal speed and demonstrated that reduction in FCCE can be achieved under varying speed environments, as also concluded by Poonthalir and Nadarajan [16]. Moreover, in existing FCCE models, the basic distinction between the two groups (i.e., micro and macro views, see Section 2.2) is the simulated model of vehicle speed (i.e., constant or non-constant). From the perspective of transportation activities, three factors influence vehicle speed, including traffic conditions, driver behaviors, and speed limits. Traffic conditions are characterized by traffic flow, especially during rush hour. This factor includes a step function to simulate varying speed, such as the functions proposed by Kuo [17], Kazemian and Aref [18], and Mirmohammadi et al. [19]. Driver behaviors is the main factor affecting gear selection, acceleration/deceleration, and idle control. Poonthalir and Nadarajan [15] used triangular distribution to simulate vehicle speed fluctuation between a minimum and maximum speed and stated that vehicles travel at several “most likely” speeds, indicating that a driver may select chronic gears at these speeds to complete transportation activities. Speed limits are an especially important factor on city roadways. Koc et al. [1] proposed speed zones to describe speed limitations according to real traffic roadways, with each zone having a speed limit and nested nature. Leng et al. [2,4] also applied speed zones, named “regional” representation.

Among the various factors affecting FCCE, fleet size and type are also important in most distribution activities [1,20,21,22]. Koc et al. [21] illustrated the benefits of a mixed fleet in reducing CE. Koc et al. [1] also demonstrated that a mixed fleet could reduce logistics costs and capacity utilization rates. Pitera et al. [23] explored rules-of-thumb for vehicle assignment within a mixed fleet to clarify simple implementations, such as assigning cleaner vehicles to routes with more clients and longer travel distances. Xiao et al. [24] emphasized that mixed fleets are concerned with individualized features including fleet types, CE rates/models, and load capacities.

### 2.2. Research about FCCE Models

Demir et al. [13] state that transportation activities can have damaging on environmental and public health by producing harmful emissions. Thus, accurate estimation models are required to measure and reduce FCCE during planning. To date, a variety of different analytical estimation models have been developed. According to Demir et al. [14] classified, three increasingly complex model types have been proposed, as described in Table 1:

The above three model groups are categorized by complexity. Demir et al. [14] also discussed load (power)-based and regression-based emission models. The former investigates vehicle gross weight and vehicle-specific parameters, such as fuel type, engine, and size. The latter considers prediction models by statistically analyzing the relationship between FCCE amount and its factors. For example, the Japanese government [38] indicated that travel distance per volume unit of fuel used is strongly correlated to vehicle gross weight, which forms the basis of the FCR model. Among the above three model types, FM/Micro is simplified/complicated version of Macro, and the nature of conversion is available between FM/Micro and Macro. Actually, from an instantaneous estimation perspective, FM is a part of Macro, because the parameters in FM, namely vehicle-specific and travel speed parameters, are negligible or constant. Therefore, the model types can be classified into non-instantaneous and instantaneous models. From the perspective of accuracy for estimating FCCE, Micro views are the best, followed by Macro, and then FM. To better understand FCCE models, please see Lin et al. [12], Demir et al. [14], and Demir et al. [13].

### 2.3. Research Concerning LCLRP

Environmental and public health require the sustainable development of the environment, economy, and society. Low-carbon supply chain network design has become an important area of research [39]. As an essential tool in the supply chain, the LRP should consider reduction of FCCE. Various researchers have analyzed and modeled the LCLRP and its variants:

- Mohammadi et al. [40] proposed a biobjective LCLRP by optimizing logistics costs and traveling distance (i.e., low carbon/green objective). Although traveling distance is a significant factor in FCCE, other parameters, especially vehicle-specific parameters and load, should be considered in the model;
- Govindan et al. [41] presented a more detailed model by combining traveling and fixed CE of depots and manufacturers, with several coefficient matrices (e.g., uniform distribution) used to consider the effect of vehicle and load. However, these coefficient matrices are generated with uniform distribution, which does not accurately reflect FCCE; This approach was also applied in Chen et al. [39] and Nakhjirkan and Rafiei [42];
- Validi et al. [43,44,45] used fuel efficiency and distance to calculate the FCCE, like Kuo et al. [17] and Poonthalir and Nadarajan [16]. Faraji and Afshar-Nadjafi [46] proposed a modified method considering the extra fuel consumption caused by carrying each extra loads. Tang et al. [47] applied the method to calculate routing CE by giving parameters for each edge, with the CE of depots/inventory also considered;
- Qazvini et al. [48] proposed a SOM considering the cost of fuel consumption as a constraint. Although this method is effective, it is more appropriate to view the FCCE cost as a traveling cost in the real world;
- Koc et al. [1] considered a city in which goods need to be delivered from a depot to clients located in nested zones characterized by different speed limits and used CMEM to estimate FCCE, which was considered a traveling cost. This followed Leng et al. [2], who studied an extensive version of the RLCLRP using a shared mechanism-based hyperheuristic. Leng et al. [4] also modeled a biobjective RLCLRP tackled by quantum-inspired MOHH;
- Rabbani et al. [49] developed a MOM for the LCLRP with a heterogeneous fleet. Among the three objectives, cost, distance, and CE were viewed as contradictions. Fuel consumption rate, similar to Pourhejazy et al. [5], was used;
- Toro et al. [50] deduced the FCCE model by analyzing the forces acting on a vehicle and found the model to be remarkably similar to the CMEM. In their MOM, they also looked at fuel consumption and total emissions associated with the fuel consumption model;
- Wang and Li [51] applied the FCR considering road slope as a FCCE model. However, in their MOM, somewhat confusingly, both objectives were cost objectives, and penalty and vehicle fixed costs were added with FCCE cost;
- Zhang et al. [3] applied the FCR model to calculate CE and used the quantum evolutionary algorithm to solve the proposed model. Wang et al. [30] subsequently developed an ant-based hyperheuristic to solve the model by Zhang et al. [3]. Leng et al. [28] proposed an extensive version of the model by Zhang et al. [3], and a quantum-inspired hyperheuristic to solve it. Zhao et al. [29] also developed an integrated model for the LCLRP by defining the FCCE cost, and developed an evolutionary hyperheuristic to solve it; Qian et al. [52] modified the model by Zhang et al. [3] with a biobjective model, and used tabu search-based MOHH to solve it;
- Wang et al. [27] developed a SOM using the FCR to estimate FCCE and as a part of costs.

From the above LCLRP-based papers, four methods were used for estimating FCCE: i.e., distance-based [39,40,41,42], fuel efficiency and distance-based [5,43,44,45,46,47,48,49], load and distance-based [3,27,28,29,30,52], and microscopic [1,2,4,50]. In addition, five modes of processing FCCE can be summarized: SOM using FCCE amount [3,28,30], SOM using FCCE as a cost [1,2,4,27,29,42], SOM using FCCE as a constraint [48], MOM using FCCE as an objective [39,40,41,43,44,45,46,47,49,50,52], and MOM using FCCE as a cost [4]. With continued development and attention, the LRP with consideration of environmental effects will become a focus of future operational research. However, in the real-life supply chain, FCCE cost should be added as a traveling cost, which also should include road tolls and parking fees, as per Koc et al. [1] and Leng et al. [2,4,29]. Therefore, we applied the same method [4] and defined FCCE as a traveling cost as the first objective (economic and environmental effect), delivery duration (i.e., total traveling time) as the second objective, and client satisfaction (social effect) (i.e., total client waiting time) as the third objective. For the solution approach, we reviewed recent literature regarding MOHH in the following section.

### 2.4. Research about MOHH

Metaheuristics have been widely tailored for different domains. However, selection of the best algorithm and configuration of parameters and operators/pairs to solve problems can be difficult and time-consuming [53,54]. Moreover, even though a single tailored-search solution may achieve good performance, it is impossible to solve all cases with high efficiency. Therefore, the definition of hyperheuristic emerges while in such situations. Walker et al. [55] claimed that hyper-heuristic approaches are gaining renewed interest due to the development of more applicable search methods. The raw hyper-heuristic ideal was initially derived by Denzinger et al. [56], with the basic concept further developed by Cowling et al. [57] as “heuristics to select heuristics”. Burke et al. [58] then proposed a comprehensive definition including (1) heuristic selection and (2) heuristic generation. The difference between heuristic selection and generation is recognized by the initial solutions; the second applies LLH to generate complete solutions beginning with empty solutions, whereas the first is initialized with complete solutions.

Metaheuristics are domain-specific, fine-tuned, and tailored-made rules-of-thumb that provide a set of mechanisms to search the domain space directly with the goal of obtaining optimal or near-optimal solutions. However, the purpose of hyperheuristic is to improve the level of generality and portability by selecting/generating appropriate operators (i.e., control operator space) to optimize rather than directly modify the domain-specific space. In other words, compared with customized methods, hyper-heuristic approaches are generally applicable to other problem domains as they contain no domain knowledge. Although Kumari and Srinivas [59] demonstrated that domain-specific, fine-tuned, and tailored-made methods will unquestionably outperform a general framework, hyperheuristics sharing the same/similar operators would also obtain good, possibly better solutions [2,28,29,30,53].

The framework of the selection hyperheuristic involves HLH and LLH. The former searches the space formed by a set of LLHs, which directly optimize the space of domain-solutions [60]. Two decisions are defined in HLH: i.e., selection strategy and acceptance criterion. Selection strategy controls and monitors the performance of each LLH to intelligently choose the appropriate operators, with a single candidate solution then generated. After that, the new solution is either accepted or rejected. For LLH, all domain-specific knowledge is provided, including encoding, decoding, chromosome, and heuristics information, such as operators, crossovers, mutations, local searches, or metaheuristics [60]. It is worth noting that, a domain-barrier is developed to prevent domain knowledge from LLH to HLH; however, HLH is allowed to access problem domain-independent information, such as number of operators, count/time of applied operators, fitness rate improvement, and fitness/objective values.

To put the selection strategy into perspective, three main types have been observed according to the source of feedback information: (i) online-learning, which occurs when a hyperheuristic is performed and includes choice function (CF) [2,4,28,57], multi-armed bandit [2,4,28,53,54,55], ant-based selection [29,30], and quantum-inspired selection (QS) [4,28,29]; (ii) offline-learning, which applies rules or programs to gather information by training a set of instances, including learning classifier systems, case-based reasoning, and genetic programming; and (iii) no-learning, which does not use any feedback information from search space, including simple random (SR), random descent, and random permutation. In contrast, there are two main types of acceptance criterion: (i) deterministic acceptance, which applies a 0–1 method-based strategy, including all moves (AM), improving and equal, and only improving; and (ii) nondeterministic acceptance, which calculates acceptance probability to judge the acceptance of new solutions, including simulated annealing, great deluge acceptance (GDA) [61], and late acceptance (LA) [61]. For a comprehensive understanding of hyperheuristics, please see Chakhlevitch and Cowling et al. [62], Burke et al. [58,63]. 

Although hyperheuristics have mainly been applied in SOMs, such as VRP [55], LRP and its variants [2,28,29,30], and sales summit [57], many studies have utilized hyperheuristics to tackle MOPs. For brief review of MOHH before 2014 see Maashi et al. [64], Table 2 provides a list of MOHH applications in MOPs since 2014, including the source of publication, published year, domain-problem, and main characteristics/components.

Based on the development of MOHHs in Table 2, LLHs can be operators and metaheuristics, and thus two main modules can be obtained: (1) MOEA-based hyperheuristic (MOHH-I) which utilize operators (i.e., crossover, mutation, and domain-specific operators) or components (such as leader selection methods and archiving strategies in MOEAs [65,67,68,79]) as LLHs and corresponding elitism selection strategies, such as NSGA-II ranking mechanism [54,57,59,60,74,78,81,82] and Pareto Strength (SPEA2) [57,78], as acceptance criteria. Here, HLHs can access the objective value [60] and the methods involving objective space instead of solution space are independent of the domain problem. (2) Basic MOHH-II, which use MOEAs as LLHs, e.g., Li et al. [60,83] and Maashi et al. [61,64], and design corresponding acceptance criterion, such as AM [61,64], improving and equal [67,68,79], GDA and LA [64,83]. However, this classification is not shared by other studies.

MOHHs have been successfully developed in both continuous and combinational optimization problems (“domain-problem” column in Table 2). To the best of our knowledge, only our previous studies have applied MOHHs to tackle the MORLCLRP, that is, Qian et al. [52] and Leng et al. [4]. Regretful, no other researcher has yet applied MOHHs to solve MOMs of the LRP, or VRP. The reasons for this may be as follows: (i) compared with other domains, much more time and effort are needed for programing effective LLHs of the MORLCLRP; (ii) greater computing time is required to execute the MORLCLRP algorithms; and (iii) the MORLCLRP is a fairly complex NP-hard problem, which means that it can be difficult to achieve a good Pareto set. Therefore, corresponding operators and metaheuristics for the MORLCLRP are urgently needed.

## 3. Mathematical Model

The applied model of estimating FCCE amount is the CMEM, and the corresponding parameters in the CMEM can be obtained from Ref. [1,2]. The descriptions and assumptions of domain problem are given in Section 3.1; Section 3.2 conducts the corresponding mathematical formulation and the necessary constraints; finally, we also provide some valid but unnecessary restrictions on the MORLCLRP in Section 3.3.

### 3.1. Description and Assumption of MORLCLRP

The MORLCLRP can be described on a complete and directed graph *Ω* = (*N*, *E*) with a vertex set *N* and an edge set of *E*. *N* consists of a subset of *D* of *N_d_* candidate depots and a subset of *C* = *N*\*D* of *N_c_* clients. Each client *c*∈*C* has a non-negative pickup demand *p_c_* and delivery demand *d_c_*, to be served exactly once, and is assigned to a single depot *d*∈*D* with capacity *w_d_* and renting cost *FD_d_*. The shipment of clients demand from the chosen depot is carried out by an unlimited set *V* = {*v*_1_, *v*_2_, *v*_3_} of heterogeneous vehicles with capacity *Q_v_* and one-time renting cost *FV_v_*; and traveling fuel consumption amount *W_ijv_* of each edge (*i*, *j*)∈*E* = {(*i*, *j*): *i*, *j*∈*N*, *i*≠*j*}\{(*i*, *j*): *i*,*j*∈*D*} is calculated by the CMEM model, and it is worth noting that the distance of each edge (*i*, *j*)∈*E* are obtained by taxicab geometry (see Koc et al. [1] and Krause [84]). 

The following are the hypotheses made: (1) each client is visited only once by a single vehicle and depot; (2) each vehicle must return the original depot from where it departs; (3) the load of each vehicle must be less than its vehicle capacity in service time; (4) the depot must serve the clients assigned to it; and (5) the vehicle must arrive at each node before the closing time windows.

### 3.2. Mathematical Model

To formulate the problem, we define the following additional decision variables. Let *x_ijv_* be equal to 1 if a vehicle of type *v*∈*V* travels on edge (*i*, *j*)∈*E* and to 0 otherwise; let *y_j_* be equal to 1 if a depot *j*∈*D* is selected and to 0 otherwise; let *z_ij_* is equal to 1 if client *i*∈*C* is serviced by depot *j*∈*D* and to 0 otherwise. A multiobjective formulation of MORLCLRP is given by:

(1) $$\min\;f_{1}=\sum_{j\in D}FD_{j}y_{j}+\sum_{i\in C}\sum_{j\in D}\sum_{v\in V}FV_{v}x_{ijv}+\left(f_{FC}+\zeta f_{CE}\right)\sum_{i\in N}\sum_{j\in N}\sum_{v\in V}W_{ijv}x_{ijv}$$

(2) $$\min\;f_{2}=\sum_{i\in C}\sum_{j\in D}\sum_{v\in V}AT_{j}x_{ijv}$$

(3) $$\min\;f_{3}=\sum_{j\in N}\sum_{i\in C}\sum_{v\in V}\left(max\left\{l_{i}-AT_{i}-ST_{i},0\right\}\right)x_{jiv}$$

where *f_FC_* and *f_CE_* are the unit price of 1 L of fuel and 1 kg of CE, respectively; *AT_j_* is the time which service starts at node *j*∈*N*. Objective (1) is to minimize the total costs consisting of opened depots, vehicle, FC, and CE costs, corresponding to the economic and environmental effects, and the reason of simultaneously using FC and CE is that they represent the costs of consuming fuel and purchasing carbon emission from carbon trading market. Objective (2) aims at optimizing the total service time, corresponding to the long-term benefit. And the clients’ satisfaction is modeled by the total client waiting time in objective (3), corresponding to the social interest. The constraints (4–23) are the necessary restrictions for the MORLCLRP.

The following two are degree restrictions, in particular constraint (4) makes sure that each client must be visited only once, and constraint (5) ensures that entering and leaving arcs to each node are equal:

(4) $$\underset{j\in N}{\sum }\underset{v\in V}{\sum }x_{ijv}=1,i\in C$$

(5) $$\underset{j\in N}{\sum }\underset{v\in V}{\sum }x_{jiv}=\underset{j\in N}{\sum }\underset{v\in V}{\sum }x_{ijv},i\in C$$

Constraints ((6) and (7)) ensure that each client must be visited by a single depot and vehicle:

(6) $$\underset{j\in D}{\sum }z_{ij}=1,i\in C$$

(7) $$x_{ijv}+\underset{k\in N}{\sum }\underset{p\in V,p\neq v}{\sum }x_{jkp}\leq 1,i\in N,j\in C,v\in V$$

Restrictions ((8)–(10)) define illegal routes, i.e., each vehicle must return back to the original departure depot:

(8) $$\underset{v\in V}{\sum }x_{ijv}\leq z_{ij},j\in D,i\in C$$

(9) $$\underset{v\in V}{\sum }x_{jiv}\leq z_{ij},j\in D,i\in C$$

(10) $$\underset{v\in V}{\sum }x_{ijv}+z_{ik}+\underset{m\in D,m\neq k}{\sum }z_{jm}\leq 2,k\in D,i,j\in C$$

The above three restrictions have been proved by Karaoglan et al. [85]. Constraints (11) ensures that total demand supplied/picked up by a depot cannot exceed its capacity; constraints (12) and (13) are the extra bounds about the load when all vehicles depart from and return back to the depot:

(11) $$max\left\{\underset{i\in C}{\sum }p_{i}z_{ij},\underset{i\in C}{\sum }d_{i}z_{ij}\right\}\leq w_{j}y_{j},j\in D$$

(12) $$\underset{j\in C}{\sum }\underset{v\in V}{\sum }L_{ijv}=\underset{j\in C}{\sum }d_{j}z_{ji},i\in D$$

(13) $$\underset{j\in C}{\sum }\underset{v\in V}{\sum }L_{jiv}=\underset{j\in C}{\sum }p_{j}z_{ji},i\in D$$

where the *L_ijv_* is the dynamic load of a vehicle of type *v*∈*V* traveling over an edge (*i*,*j*)∈*E*. The dynamic load of each vehicle must not exceed the its capacity, which is ensured by constraint (14); the relaxed (if *d_j_* < *p_j_*) or intensified (if *d_j_* > *p_j_*) is given in restriction (15); constraint (16) implies that the delivery and pickup demand of each client is satisfied; constraints (17) and (18) indicate that the load of vehicle of type *v*∈*V* must be equal to the total delivery/pickup demand when departs from/return back to the depot:

(14) $$0\leq L_{ijv}\leq Q_{v}x_{ijv},i,j\in N,v\in V$$

(15) $$\left(d_{j}-p_{j}\right)x_{ijv}\leq L_{ijv}\leq \left(Q_{v}-d_{j}+p_{j}\right)x_{ijv},i\in N,j\in C,v\in V$$

(16) $$\underset{i\in N}{\sum }\underset{v\in V}{\sum }\left(L_{ijv}-d_{j}\right)x_{ijv}=\underset{i\in N}{\sum }\underset{v\in V}{\sum }\left(L_{jiv}+p_{j}\right)x_{ijv},j\in C$$

(17) $$\underset{i\in D}{\sum }\underset{j\in C}{\sum }L_{ijv}=\underset{i\in C}{\sum }\underset{j\in N}{\sum }d_{i}x_{ijv},v\in V$$

(18) $$\underset{i\in C}{\sum }\underset{j\in D}{\sum }L_{ijv}=\underset{i\in C}{\sum }\underset{j\in N}{\sum }p_{i}x_{ijv},v\in V$$

The next two are used to restrain the vehicles’ behavior according to clients’ time windows:

(19) $$AT_{j}=\left(max\left\{AT_{i},e_{i}\right\}+ST_{i}+TT_{ij}\right)x_{ijv},i\in C,j\in N,v\in V$$

(20) $$0\leq AT_{i}\leq l_{i},i\in C$$

where *ST_i_* corresponds to the service time of node *i*∈*C*; *TT_ij_* is the travel time over an edge (*i*,*j*)∈*E.* (*e_i_*, *l_i_*) represents the time windows of client *i*∈*C*. Looking at constraint (19), if a vehicle arrives at client *i*∈*C* before time *e_i_*, it waits until *e_i_* to start to serve the client. Constraint (20) is designed for assumption (5). The next inequality is to restrict that opened depots must serve clients:

(21) $$z_{ij}\leq y_{j},i\in C,j\in D$$

(22) $$\underset{i\in C}{\sum }z_{ij}\geq y_{j},j\in D$$

Constraint (21) ensures that an unselected depot mustn’t service the clients; constraint (22) makes sure that an opened depot must serve clients. The above constraints are significant for the proposed problem, and we introduce some extra valid restrictions but not necessary in Section 3.3.

### 3.3. Other Valid Constraints

We introduce several polynomial size valid inequalities for our problem, which were used by several studies for VRPs/LRPs. The first inequality is the subtour elimination:

(23) $$x_{ijv}+x_{jiv}\leq 1,i,j\in C,v\in V$$

Constraint (23) derived from Koc et al. [22] are only suitable for the directed graph with specific constraints, such as time windows and simultaneous pickup and delivery, instead of the basic LRP and heterogonous fleet. The next inequality is to restrict the opened depots and vehicles:

(24) $$\underset{j\in C}{\sum }x_{ijv}\leq y_{i},i\in D,v\in V$$

(25) $$\underset{i\in C}{\sum }\underset{v\in V}{\sum }x_{ijv}\geq y_{j},j\in D$$

Constraint (22) ensures that an unselected depot mustn’t service the clients; constraint (23) makes sure that an opened depot must serve client; constraint (24) guarantees that an unselected depot cannot assign vehicles; constraint (25) imposes that an opened depot must have vehicles to serve clients. The third restriction is introduced to the limitation of vehicles:

(26) $$\lceil \frac{max\left\{\sum_{i\in C}d_{i},\sum_{i\in C}p_{i}\right\}}{\underset{v\in V}{max}\left(Q_{v}\right)}\rceil \leq \underset{i\in D}{\sum }\underset{j\in C}{\sum }\underset{v\in V}{\sum }x_{ijv}\leq N_{c},$$

Constraint (26) provides a lower and upper bound on the number of vehicles originating from depots, and ⌈•⌉ is the smallest integer larger than •. The fourth valid inequity is the number of opened depots:

(27) $$\underset{i\in D}{\sum }w_{i}y_{i}\geq max\left\{\underset{i\in C}{\sum }d_{i},\underset{i\in C}{\sum }p_{i}\right\}$$

Constraint (27) states that the total capacity of opened depots is larger than the maximum demand. However, this restriction is not necessary, since sometimes if it is met, but it cannot provide restriction. The classical example is the Perl83-55×15 in Barreto et al. [86].

(28) $$\underset{j\in C}{\sum }x_{ijv}+\underset{j\in C}{\sum }x_{jgv}\leq 1,i,g\in D,i\neq g,v\in V$$

The final valid inequity is a complementary for constraint (10), which forbids the different depots in a single route. Looking at the extra valid restrictions, most of them can be expressed by the constraints (4–23). However, the aim of this section is to help readers easily understand the proposed problem.

## 4. Proposed Method

This section describes the proposed MOHH from two aspects: (1) domain-specific level and (2) high-level strategy. Section 4.1 introduces the necessary chromosome representation, a general framework for MOEAs as LLHs, and practical operators. Section 4.2 provides the MOHH framework for solving the proposed MORLCLRP. Section 4.3 discusses the four selection strategies. Section 4.4 discusses the three acceptance criteria.

### 4.1. Domain Method

#### 4.1.1. Chromosome Representation

In the proposed algorithm, the chromosome represents a complete solution, i.e., a collection of routes. Each route is stored in the cell array, i.e., *F* = {*f*_1_,*f*_2_,…,*f_k_*}, where *f_i_* is a complete vehicle route with opened depots inserted at its two ends. We also provide related information on the effects of vehicle and traveling on the objectives, such as starting/returning load, type of vehicle, traveling cost, time, and client waiting time in the attribute array, similarly to the solution representation proposed by Leng et al. [4].

Furthermore, a population-based search is used in our framework, so individuals in the population are constructed by randomly selecting clients to form a “super-client” (i.e., set of clients) assigned to each vehicle. Each vehicle is randomly assigned to the depot if only constraints (4)–(23) are met.

#### 4.1.2. Applied Operators

In this section, we provide domain-specific operators, as used by Leng et al. [2] for SOMs and Leng et al. [4] for biobjective models. Here, we first study the MORLCLRP by minimizing total cost, service duration, and client waiting time. Therefore, corresponding modifications are conducted in the applied operators by calculating the three objectives. However, we also follow the classification of Leng et al. [4], i.e., mutational operator (Mu), nondominated local search (NDLS), and dominated local search (DLS). Mu provides randomness to avoid local optima, NDLS produces many nondominated solutions, and DLS accelerates the process of searching approximate Pareto solutions by finding the solutions dominating parents. For iterations, DLS can provide high performance in short-term iterations; Mu plays a role in escaping local optima in medium-term iterations; and, NDLS performs best for nondominated solutions in long-term iterations. The corresponding design can be seen in Leng et al. [4].

#### 4.1.3. General Structure of MOEAs for MORLCLRP

Algorithm 1 is the proposed general framework of MOEAs for the MORLCLRP. The proposed framework first creates an initial population (*Pop*) made of feasible random chromosomes. Afterwards, necessary parameters are set for the MOEAs, if needed. A main loop is then performed, stopping when the maximum number of generations (*max_gen_*) is reached.

In each generation, a Mu is randomly selected for the chosen individual (if random < *p_m_*) to obtain a child solution, then a local search operator is randomly chosen to optimize the obtained solution, which is merged into the parent population. Afterwards, if the size of the merged population is larger than *N*, the elitism selection strategy is applied to survive the best *N* individuals for the next generation. The algorithm ends when the main loop stops, returning the survived *Pop*.

**Algorithm 1** General framework of MOEAs for LCLRP**Input:***Pop*, *max_gen_*, etc.**Output:** child population (*Pop*)1: Generate corresponding parameters of meta-heuristics//*Main loop*2: **Repeat**3:  *i*:=*i* + 14:  **for** each solution in *Pop*
**do**     // *Mutation*5:    **if** random < *p_m_*
**then**6:     **Mu:** randomly choose a mutational operator7:     Obtain a child *C*8:    **end if**     // *Local search*9:    **Local search:** randomly select an operator from **NDLS**10:    and **DLS** to optimize Child *C*/Parent *P* (if random > *pm*)11:    Obtain a new solution *CC*12: **end for**13: Obtain child population *CP*14. **Merge population:**
*All* = [*Pop*,*CP*]15: **Update of solutions:** apply the environmental selection of    meta-heuristic to generate the Pop into next generation16: **Stopping criteria:** if stopping criteria is satisfied, then    stop and output *Pop*. Otherwise go to Step 3.17: **until**
*i* = *max_gen_*

### 4.2. Framework of MOHH

The proposed algorithm first creates an initial population (*Pop*) made of feasible random chromosomes, with certain acceptance criteria, selection strategies, and LLH parameters generated if needed. Afterwards, a main loop is implemented, stopping when the maximum number of iterations (i.e., *Max_iter_*) is reached.

For each main loop iteration, a promising MOEA is chosen by one selection strategy (i.e., SR, MAB, CF, and QS) to transform the solution space of the proposed problem, and *Cpop* is obtained. Afterwards, an acceptance criterion (i.e., AM, GDA, and LA) is performed to accept *Cpop* if the requirements of the selection strategy are met. The algorithm ends when the main loop stops, returning the approximate Pareto solutions. We also provide an archiving strategy by storing 5 × *N* nondominated solutions where the nondominated sorting and crowded distance in NSGA-II [87] are used. An overview of the pseudocode of the proposed MOHH is given in Algorithm 2.

The main differences between the algorithm in this study and that of our previous work could be identified that the credit assignment (reward value) in this paper concerns D-mtrix (D), generational distance (GD), and population dominance (PD) instead of hypervolume (HV), PD, and Spacing. The main reason is that the difference between parent and child population can be identified by the first three. HV and Spacing only represent the characteristics of the child population.

**Algorithm 2** General framework of MOHH // *Initialization*1: *Citer*:=0 (Current iteration)2: Parameter setting: parameters in Hypervolume, *Max_iter_*, *N*, etc.3: Generate population *Pop*4: Calculate the multiobjective fitness // *Main loop*5: *Cpop* = *Pop*; *Ppop* = *pop*; *ArPop* = *Ø*;6: **Repeat**7: *Citer*:=*Citer* + 18: // *High-level selection strategy*9: **Apply** SR/MAB/CF/QS to select the promising LLH *op*10: // *Low-level heuristics*11: **Apply** the selected *op*th MOEA to generate *Cpop*12: // *High-level acceptance criterion*13: **Apply** the selected acceptance criterion (GDA, LA, and AM)   to determinate the *Pop* of next iteration14: // *Archive population (if needed)*15: **Save** all nondominated individuals into *ArPop*;16: **if** number of individuals in *ArPop* is larger than 5 × *N*
**then**17:  **Apply** environmental selection of NSGA-II to remove the     individuals with much more crowded18: **end if**19: **until**
*Citer* = *Max_iter_*20: **if**
*ArPop*
**then**
21: **Apply** environmental selection of NSGA-II to remove the    4 × *N* individuals with much more crowded22: *Pop* = *ArPop*23: **end if**24: **return**
*Pop*

### 4.3. Heuristic Selection Strategies

In this study, well-known and modified heuristic selection approaches are used as selection MOHH components for choosing the appropriate MOEA at each iteration/stage.

#### Simple Random

For SR, a random LLH (MOEA) is simply chosen at each iteration. It is usually used as a baseline for comparison against the learning hyper-heuristic methods.

### 4.4. Fitness Rate Rank Based Multi-Armed Bandit (FRR-MAB)

FRR-MAB is an upper confidence bound algorithm. In MAB, the key for a successful algorithm is to find a good trade-off between exploration and exploitation, i.e., the best LLH (exploitation) or other LLH (exploration). In this mechanism, there exists a credit assignment and operator selection. 

In the process of FRR-MAB for MOM, the population dominance relationship is applied to calculate the reward of each LLH. Afterwards, the reward is stored in a sliding window, organized as a first-in-first-out mechanism. This is because, in dynamic environments, the performance of an operator in a very early stage may be irrelevant to its current performance [54]. Normalization is then applied to calculate the empirical quality estimate, and a confidence interval is obtained by the usage of operators. Finally, the credit value of each LLH is achieved. The LLH with a maximum credit value is selected for the next iteration.

#### 4.4.1. Choice Function

The CF heuristic selection method was first used by Maashi et al. [64] to tackle a series of continuous optimization benchmarks. In their CF, a two-stage ranking scheme calculates performance value, with CPU seconds elapsed since the last call to a low-level (meta) heuristic applied as the confidence internal. The final score (or value) for a given MOEA:

(29) $$CF\left(llh\right)=\alpha f_{1}\left(llh\right)+f_{2}\left(llh\right),llh\in H$$

where, *α* is a positive parameter; *H* is the pool of LLHs; *llh* is the index of an MOEA; *f*_1_/*f*_2_ is the performance value(intensification)/elapsed CPU time (in seconds) since the time *llh* was last called (diversification). In this paper, we use a simple method to calculate the reward value of *llh* at *t* iteration using D-matrix, generational distance (GD), and population dominance (PD):

(30) $$RV_{llh,t}=D\times GD\times PD.$$

where, *D* is the difference in covered area size between parent front and child front; *GD* is the generational distance; and *PD* is the nondominated reward in FRR-MAB. The LLH with the highest *CF* value is preferentially selected.

#### 4.4.2. Quantum-Inspired Selection

The QS heuristic selection method proposed in our previous work [4] uses a qubit chromosome as a probabilistic representation instead of binary and numeric ones. Population dominance was used to judge the angle direction, and a comprehensive function that merges PD, HV, and spacing was used to reward the promising *llh*. A q-gate was introduced to update the state of each qubit in individual and heuristic space. The *β* value of each LLH reflected the exploitation of an algorithm, and probability matching (roulette selection) was used to explore the solution spaces to avoid local optima. In this paper, for fair comparison, we use the same reward function in CF to reward the promising *llh*. For the corresponding design of QS, please see Leng et al. [4].

### 4.5. Move Acceptance Methods

Move acceptance methods play a key role in MOHHs by deciding to accept or reject candidate solutions produced by the chosen LLH. There are several simple and elaborate acceptance strategies (AS) used as part of MOHHs. Maashi et al. [64] initially applied AM as an AS and after developed two much more efficient ASs: i.e., GDA and LA [61] which use D-matrix to provide the dominant relationship between parent and child populations. These ASs are usually used for MOHH-II modules, which utilize MOEAs as LLHs. Elitism selection strategy can also be ASs, known as best acceptance. Leng et al. [4] and Li et al. [60,83] used nondominated sorting and crowding distance derived from NSGA-II [87] to select promising individuals for the next iteration. In this paper, we consider three well-known ASs: i.e., AM, GDA, and LA, as they performed well in Li et al. [60], Maashi et al. [61,64], and Leng et al. [4]. It is worth noting that the first two performs after normalizing the dimension of three objectives in this paper.

## 5. Computational Evaluation

### 5.1. Implementation Aspects and Configuration of Parameters

The MOHH was coded in parallel in MATLAB 2018a (v9.4.0.813654) using a 4.0 GHz Intel Core i7-6700K with 12 GB of RAM and running Windows 10; it is embedded in the CLOR tool implemented by the MATLAB platform (available via email to the authors).

We followed the parameter configurations suggested by Leng et al. [4]. Here, the maximum iteration (*max_iter_*) was 100 for the outside loop. The maximum number of generations depended on the size of each instance:

*Max_gen_* = *α* × (*N_c_* + *N_d_* + *V_max_*) (31)

where *N_c_*(*N_d_*) is the number of clients (depots); and *V_max_* is the maximum number of vehicles with minimum capacity. Multiplier *α* depended on the size of the instance and objectives, and was 45 for 20 clients, 57 for 30 clients, 68 for 40 clients, and 80 for 50 clients.

The size of the population and archiving population were set at 100 and 500, respectively. The mutation rate *p_m_* was set to default (0.35), as per Leng et al. [4]. For parameters in the acceptance criteria, we applied the default values from Maashi et al. [61] and Leng et al. [4], i.e., rain speed (*S* = 3 × 10^−4^) and *C* length (*L* = 5).

For the test suite, we applied the instances used by Leng et al. [4] for the biobjective RLCLRP. We generated other sets to analyze the effects of each speed zone area on key performance indicators, as described in Section 5.2.

### 5.2. Performance Metrics

We utilized three well-known performance metrics to evaluate the performance of algorithms and problems by performing ten runs: inverted generated distance (IGD), HV, and the ratio of nondominated individuals (RNI).

The *IGD* describes the quality and uniformity of an approximate Pareto front (AF) by measuring the distance between *AF* and real Pareto front (PF), the smaller the *IGD* value, the better the distribution and convergence. The *HV* measures the covered space size between the *AF* and a reference point. The larger the *HV* value, the better the diversity and distribution. The last one is simply measured by putting together the nondominated solutions found by algorithms and the ratios between non-dominated solutions are reported. The larger the *RNI*, the better quality of *AF*.

However, as the MORLCLRP was first solved in this paper, if *PF* was unknown, we used all *AF* to form *PF*. The set *PF* was, in fact, an approximation of the real front. It is worth noting that the first two metrics were calculated after normalization.

The first hypothesis of this paper is that the use of hyperheuristics leads to better results than traditional MOEAs. The second is to analyze the effects of domain-specific parameters on the above three quality indicators. Considering these, our experiments were guided by the following research questions: (1) How do the results produced by the MOHH compare with traditional algorithms? and (2) what are the behaviors of the problem parameters affecting quality indicators?

To answer the aforementioned questions, we first applied eight MOEAs used in related work: nondominated sorting genetic Algorithm-II (NSGA-II) [87], strengthen Pareto evolutionary algorithm2 (SPEA2) [88], bi-goal evolution (BiGE) [89], nondominated sorting and local search (NSLS) [90], grid-based evolutionary algorithm (GrEA) [91], indicator-based evolutionary algorithm (IBEA) [92], NSGA-III [93], region-based selection in evolutionary-II (PESA-II) [94], then applied the three best-performing MOEAs as LLHs for MOHHs, followed by the next three well-performing MOEAs as LLHs. The results were evaluated using quality indicators. From the general questions, we will provide purposes in each experiment.

### 5.3. Efficiency of MOHH

Although Kumari and Srinivas [59] stated that the efficiency of general framework methods is inferior to problem-specific solvers incorporating domain-specific knowledge and fine-tuned, tailor-made strategies, we compared our proposed MOHH and other MOEAs by analyzing the IGD and HV values by solving different instances. Table 3 and Table 4 provide the mean IGD and HV values obtained by the eight MOEAs and two MOHHs with different LLHs, as mentioned above. Note that values in Table 3 and Table 4 is multiplied 100 to save space.

As shown in Table 3, only SPEA2 and BiGE obtained the best mean IGD for L20-1 and L50-1, respectively. Similarly, although both obtained four third places, SPEA2 outperformed BiGE as the number of third places was larger than that for BiGE. As also seen in Table 3, QS1 using SPEA2, BiGE, and NSLS as LLHs achieved ten best IGD means compared with the first eight MOEAs and QS2 using NSGAII, NSGA-III, and PEAS-II as LLHs. Moreover, QS2 showed inferior performance to SPEA2 and BiGE, but was superior to others. Additionally, the MOHHs using archive methods outperformed the others as the mean IGD was the lowest among all algorithms, QS1a obtained nine best mean IGDs, whereas QS2a only achieved three.

Table 4 displays the mean HV for all instances. Similarly, the performances of BiGE and SPEA2 were the best among the first eight MOEAs, especially SPEA2, which achieved two first places, three second places, and six third places. NSGAII also obtained one first place. Looking at the performance using the hyper-heuristic framework, QS1 claimed first place by achieving eight first places, three second places, and one third place in 12 instances; QS2 showed lower performance achieving only three second places and two third places. The archiving method thus improved the performance of MOEAs as stated in Zhang et al. [95].

**Figure 1:**
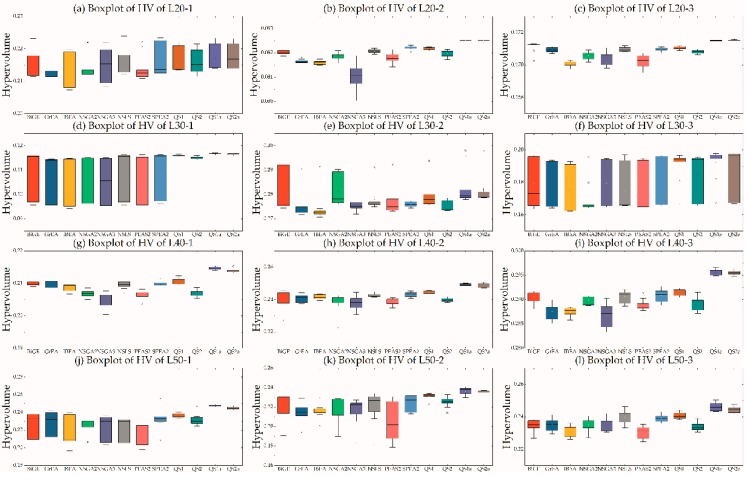
Boxplot of hypervolume of 12 MOEAs.

Figure 1 is a boxplot of hypervolumes obtained by performing 12 MOEAs. The following conclusions were drawn: (1) A single MOEA cannot outperform all other MOEAs in all instances, e.g., BiGE for L50-1 and L50-3 and SPEA2 for L50-3; (2) BiGE, SPEA2, and NSLS were superior to others, and NSGA-II, NSGA-III, and PEAS-II outperformed GrEA and IBEA in most instances. We used the scoring system [96] to classify the first eight MOEAs, and found SPEA2(213) ≺ BiGE(180) ≺ NSLS(146) ≺ PEAS(93) ≺ NSGAII(92) ≺ NSGAIII(87) ≺ GrEA(79) ≺ IBEA(46), where A(*a*) ≺ B(*b*) indicates that the score *a* of A is larger than the score *b* of B and thus A dominates B. Therefore, QS1 used SPEA2, BiGE, and NSLS as LLHs and QS2 used PEAS, NSGAII, and NSGAIII as LLHs. (3) The use of hyperheuristics (i.e., QS1) outperformed others without the hyper-heuristic framework, however, QS2 was inferior to the top three MOEAs, but outperformed the other five MOEAs, suggesting that the configuration of the LLH pool is extremely significant in designing MOHHs. (4) The archiving method can significantly improve the performance of MOEAs, as shown in Table 3 and Table 4, and Figure 1; therefore, the following experiments (except Section 5.4) used the archiving method to obtain better Pareto solutions. In brief, the hyperheuristic in this paper effectively improved performance of MOEAs by assimilating the essence and rejecting the dross of MOEAs.

### 5.4. Efficiency of Pairs in MOHH

**Figure 2:**
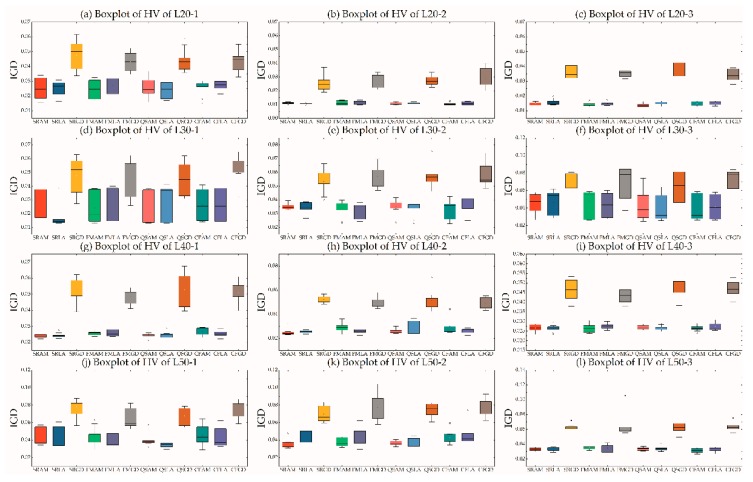
Boxplot of IGD values of 12 pairs.

**Figure 3:**
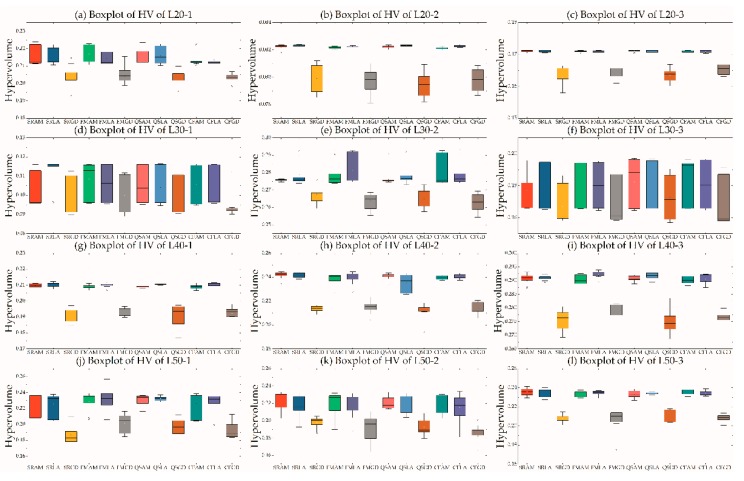
Boxplot of hypervolume values of 12 pairs.

This section compares the performance of each pair described in Section 4.2 and 4.3. Four selection strategies and three acceptance criteria formed 12 pairs, which were used to optimize the randomly generated instances. Figure 2 and Figure 3 are boxplots of the IGD and HV values, respectively. For an easy plot, we used the logogram of GDA (GD) and FRR-MAB (FM).

As shown in Figure 2, the performance of GDA was inferior to the others, even though they shared the same SSs. The performance of LA was superior to that of AM in most instances when sharing the same SS. However, different SSs sharing the same AC demonstrated different performances. For example, QSLA outperformed the others in L202, L401. A similar pattern for all pairs can be seen in Figure 3. As comparing the performance of all pairs can be difficult, we used a scoring system (see Section 5.3), with results shown in Table 5. QSLA, SRLA, QSAM, FMLA, and CFAM, were the top five pairs. The score of each SS with GDA was equal to zero, indicating that GDA was the worst AC. Conversely, LA was the best AC, and was 20 scores ahead compared with AM. The order of performance was QSLA > SRLA > QSAM > FMLA > CFAM > SRAM > FMAM > CFLA. The order of performance of AC when neglecting SS was LA > AM > GDA. The order of performance of the four SSs when neglecting AC was QS > CF > SR > FM. In the following experiments, we randomly selected one of the top three pairs in each run, i.e., QSLA, SRLA, and QSAM, to analyze the effects of domain-specific parameters on IGD and HV values.

### 5.5. Effect of Clients and Depots Locations

As each speed zone has a fixed speed, which is a significant parameter in FCCE, the locations of clients and depots are extremely important for determining which to serve. In this test suite, we randomly generated 16 instances with different depot and client locations, i.e., C*n*D*m*, *n*∈{1,2,3,*R*} and *m*∈{1,2,3,*R*}, where, *R* indicates the location of depots/clients is randomly located within a speed zone. Table 6 and Table 7 are the mean IGD and HV values of 16 pairs for each instance. Note that values in Table 6 and Table 7 is multiplied 100 to save space.

Looking at Table 6, in most instances, the mean IGD values of C1DR, C1D1, C1D2, and C1D3 were the top four allocation strategies. The worst allocation strategies were C3D1, CRD1, C3D2, and CRD2. Similar characteristics can also be derived from Table 7. We analyzed the RNI indictors in a “real” Pareto set, and found the sequence: C1D1(29.6%) > C1DR(21.6%) > C1D3(19.5%) > C1D2(13.3%) > C2D3(6.0%) > C2D2(4.0%) > C2DR(3.1%) > C3D3(1.6%) > CRD3(5.2‰) > C2D1 (2.9‰) > C3DR(2.1‰) > CRD1(0.733‰) > CRD2(0.729‰) > C3D1(0.22‰) > CRDR(0.13‰) > C3D2 (0.056‰), where the numbers in parentheses are RNI values. Although the costs of depots in zone 1 were highest, the RNI was the largest among the 16 pairs. The RNI values of C1DR, C1D3, and C1D2 were also higher than other pair. In fact, the RNI values did not represent the priority order among the 16 pairs.

### 5.6. Effect of Fleet Composition

In this section, we analyzed the effects of fleet composition on Pareto front using IGD, HV, and RNI indicators. The instance sets were the same as in Section 5.3. Table 8 shows the mean IGD, HV, and RNI values for each pair. The “HF” represents heterogonous fleet. Note that values in Table 8 is multiplied 100 to save space.

**Figure 4:**
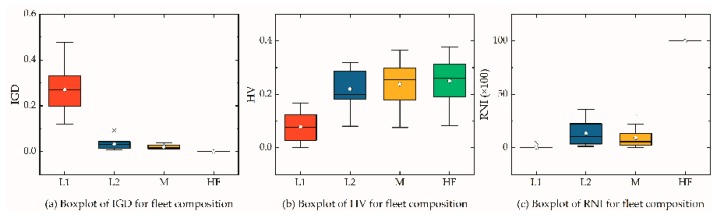
Boxplot of IGD, HV, and RNI values of each fleet composition.

As shown in Table 8, the mean IGD, HV, and RNI values of HF for each instance were better than those of L1, L2, and M. The “real” Pareto front of each instance was the same as fronts of HF. As vehicle parameters were different, the preference of distribution in each instance was also different. For example, type L2 vehicles were preferred in L20, L30, L402, and L503, whereas type M vehicles were preferred in L401, L403, L501, and L502. Therefore, as the size of instance increases, a type M vehicle will be preferred. However, the total RNI indicator of L1, L2, and M was 48.8%, lower than 50%, which demonstrates that a composited HF can reduce logistics costs, service duration, fuel consumption, carbon emissions, and client satisfaction. From the perspectives of IGD and HV values, the Pareto front of HF dominated the fronts of L1, L2, and M after removing the duplicating individuals. Figure 4 is a boxplot of IGD, HV, and RNI for each fleet composition.

### 5.7. Effect of Zones Area

Here, we estimated the effects of speed zone area on IGD, HV, and RNI values. The instances were randomly generated. Table 9 lists the IGD, HV, and RNI of each instance, where EQ/Do/TF/FF/ORI represent equal/two-fold/three-fold/four-fold/original from Koc et al. [1]. Note that values in Table 9 is multiplied 100 to save space.

**Figure 5:**
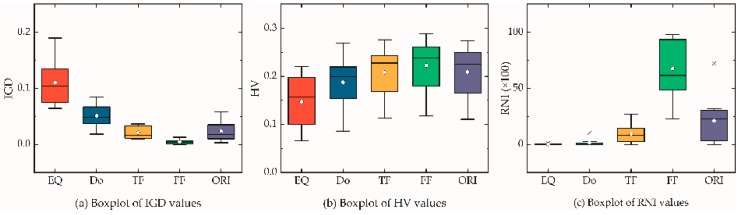
Boxplot of IGD, HV, and RNI values of each area size.

Figure 5 is a boxplot of IGD, HV, and RNI values for each area ratio. As seen in Table 9, as the ratio of the zone area increased, performance also increased, especially FF. The ratio of FF obtained the best performance, except for IGD/RNI values of L501 and HV values of L401, L501, and L502. The ratio of the ORI area zone was zone1 (9%): zone 2 (25%): zone 3 (64%). An ideal ratio for speed zone area for specific instances was shown to exist. As shown in Table 9, ORI outperformed FF in L501, which indicates that the best ratio may exist between ORI and FF; however, the best ratio is extremely hard to find as infinite pairs exist and it may change with the nature of instances.

### 5.8. Management Implications

**Figure 6:**
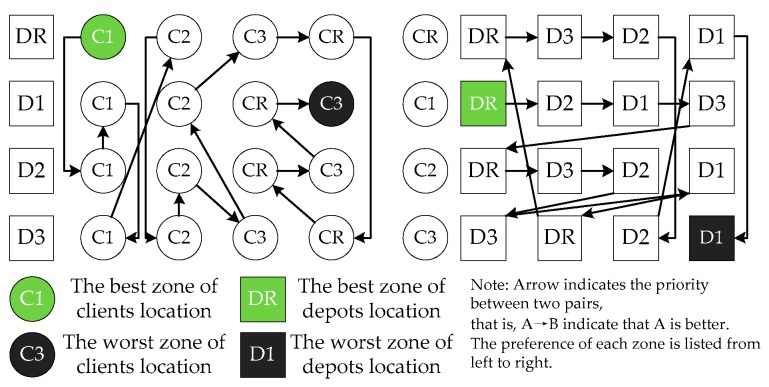
Preference of clients and depots locations.

From a management point of view, several management implications can be obtained from the results. Section 5.5 analyzed the joint effects of depot and client location. The depot and client preference in each zone are shown in Figure 6. From the perspective of depots in each zone, the clients in zone 1 were preferred for the DR, indicating that the randomly located depots (zones 1, 2, and 3) should be given preference to serve the clients in zone 1 instead of other zones. All depots located in each zone preferred to serve clients in zone 1, indicating that the depots in D2 and D3 preferred to serve clients in C1 instead of C2 and C3, respectively. From the perspectives of clients, the first three groups (i.e., CR, C1, and C2) preferred DR for service rather than the depots in D1 and D2. However, the clients in zone 3 preferred the depots in zone 3 instead of DR. Moreover, the clients in CR, C1, C2, and C3 preferred depots in D3, D2, D3, and DR, respectively. The worst allocation strategy was clients in zone 3 served by depots in zone 1. The priority was: C1DR < C1D2 < C1D1 < C1D3 < C2DR < C2D3 < C2D2 < C3D3 < C2D1 < C3DR < CRDR < CRD3 < CRD2 < C3D2 < CRD1 < C3D1. The main reasons for these results are: (1) Zone-specific parameters: i.e., speed and geographical factors. Each zone had a different vehicle speed affecting FCCE amount, and the size of each zone was also another affecting parameter; (2) Domain-specific parameters. The cost of depots in each zone was the most significant factor determining the selection of depots to serve clients. Therefore, logistics companies should analyze the effect of client and depot distribution on Pareto solutions, as the calculation of costs, service duration, fuel consumption, carbon emission, and client satisfaction are based on client and depot locations.

Section 5.6 analyzed the effects of fleet composition. From the results, we strongly conclude that the fleet composition consisted of HF can provided reduction in logistics costs, service duration, fuel consumption, carbon emission, and client satisfaction. Section 5.7 analyzed the effects of speed zone area, and we found that a best ratio range may exists for the speed zones and should be analyzed and determined, which is important for city planning decided by the economy and population development. Moreover, it is important for logistics enterprises to decide whether or not to authorize other logistics (such as local logistics companies) with a low entrust cost instead of building or renting the facilities (depots and vehicles) with a higher fixed cost.

In the experiments, we analyzed the efficiency of the proposed general framework, i.e., MOHH. Results showed that the proposed algorithms effectively tackled the proposed problem and outperformed several well-known MOEAs. Moreover, we analyzed the effects of domain-specific parameters, such as depot and client locations, fleet composition, and speed zone area on the Pareto solution indicators, i.e., IGD, HV, and RNI. We also provided several management and service suggestions to help reduce total costs (including FCCE cost) and service duration, as well as increase client satisfaction.

## 6. Conclusions

In this paper, we presented a MOHH algorithm to solve a MORLCLRP considering simultaneous pickup and delivery, hard time windows, and heterogeneous fleets. In the problem domain, we modeled a multiobjective mathematical formula, which simultaneously minimized service duration, client waiting time, and total logistics costs, where the latter was defined with respect to total costs of renting depots and vehicles as well as FCCE. In the algorithms, we proposed a MOHH, with simple random, fitness rate rank-based multi-armed bandit, choice function, and quantum-inspired selection as the high-level strategy, and all moves, great deluge, and late acceptance as acceptance criteria. Moreover, we provided a general framework of MOEAs used to perform comparative analysis.

In regard to the study aims, we: (1) verified the efficiency of the proposed algorithms; (2) comparatively analyzed the performance of 16 pairs; and (3) analyzed the effects of domain-specific parameters on performance indicators. The first experiment verified our proposed algorithms compared with eight well-known MOEAs. The second experiment showed that the performances of QS-LA, SR-LA, and QS-AM were the best among the 16 pairs. The third experiment evaluated the impact of problem parameters on Pareto solutions, and several conclusions could be obtained:

- Although method synthesis might promote the algorithm’s performance, the strategy is significant to choose and monitor the performance of each method. Moreover, the LLHs have strong ability in effecting the whole performance, therefore, the analysis should be conducted before constructing the pool of low-level heuristics;
- The HLHs are important for the algorithm’s performance, and the unmerited design may produce the poor performance than the simple random, such as FRR-MAB and GDA;
- The depot and client location has significant impacts on the logistics cost, client satisfaction, and service duration. Before determining the set of depots to open and the tracing of the routes, the joint effects of the depot and client location should be analyzed. In the context of this paper, the joint effect can be obtained: C1DR > C1D2 > C1D1 > C1D3 > C2DR > C2D3 > C2D2 > C3D3 > C2D1 > C3DR > CRDR > CRD3 > CRD2 > C3D2 > CRD1 > C3D1 (priority sequence). Moreover, we also analyzed the preference of client and depot in each speed zone, and we found that DR/D3 (C1→C2→C3→CR), D1/D2(C1→C2→CR→C3), CR/C2(DR→D3→D2→D1), C1(DR→D2→D1→D3), and C3(D3→DR→D2→D1), where A(B→C) indicates that the preference of A is B and C, and B is better than C;
- The fleet composition is another factor effecting the logistics network. From the perspectives of the results, the heterogonous fleet could always obtain better Pareto solutions;
- The zone area effects the depot and client location, and how to partition the speed zones (i.e., the best ratio of speed zones) and determine the depot and client location, to some extents, determine the logistics network. Therefore, the government and logistics companies should optimize the speed zone area for economic, environmental, and social effects.

However, as ours is a multiobjective model, the ratio of fuel consumption and CE cost is difficult to analyze. Moreover, although hyper heuristics are oriented from the generality level, the performance of LLHs significantly effects the whole performance, therefore the future work will focus on the development of MOHH-II, and may try to improve the performance of multiobjective hyperheuristics by designing more efficient high-level strategies. we may also consider the uncertainty related to the input parameters with fuzzy method [97], stochastic program [98,99], and practical application [6,100] to bring the problem closer to the reality.

## Figures and Tables

**Table 1:** Classification of models estimating FCCE.

Module	Characteristic	Example
Factor model (FM)	Only consider a few key factors, such as travel distance, vehicle load.	Fuel consumption rate (FCR) [3,6,25,26,27,28,29,30]; models by Pourhejazy et al. [5]
Macro view (Macro)	Average aggregate network parameters, factors don’t change over traveling time	Computer programs to calculate emissions from road transportation [31,32]; Methodology for calculating transportation emissions and energy consumption [33,34], etc.
Micro view (Micro)	More detailed information, second level, especially vehicle speed.	Comprehensive model emission model (CMEM) [1,2,4,13,15,21]; comprehensive power-based fuel consumption models [35]; vehicle specific power model [36,37]

**Table 2:** Applications of MOHH since 2014.

Authors [Reference] (Year)	Domain-Problem	Main Characteristics/Component
Koulinas et al. [65] (2014)	Resource constrained project scheduling problem	Domain-LLH; particle swarm optimization
Kateb et al. [66] (2014)	Runtime usage of MOEAs	Artificial selection of mutation for NSGAII
Maashi et al. [64] (2014)	MOEAs benchmark (WFG test suite)	NSGAII, SPEA2, MOGA; choice function; All moves
Castro and Pozo [67,68] (2014&2015)	MOEAs benchmark (DTLZ test suite)	Using leader selection methods and archiving strategies as LLH; IE acceptance; R2 indicator
Goncalves et al. [69] (2015)	MOEAs benchmark (UF test suite)	Using DE operators as LLH; MOEA/D; Tchebycheff function
Hitomi and Selva [70] (2015)	Architecture optimization problems	Dynamic MAB; using heuristic agents as LLH
Maashi et al. [61] (2015)	MOEAs benchmark (WFG test suite)	Choice function; great deluge algorithm and late acceptance; NSGAII, SPEA2, and MOGA
Qian et al. [71] (2016)	Multiobjective optimization	Using selection mechanisms, mutation, acceptance strategies as LLH
Kumari and Srinivas [59] (2016)	software module clustering	Using crossover and mutation as LLH; NSGAII ranking mechanism; reinforcement learning strategy with adaptive weights
Strickler et al. [54] (2016)	variability test of feature models	Fitness rate rank-based MAB; NSGAII ranking mechanism; crossover and mutation operators
Freitag and Hildebrandt [72] (2016)	Scheduling rules for complex manufacturing systems	Using scheduling rules as LLH; simulation-based genetic programming;
Guizzo et al. [73] (2017)	Integration and test order problem	Choice function, MAB; crossover and mutation operators; reward based-Pareto dominance
Li et al. [60] (2017)	Wind farm layout problem	Random, fixed sequence, choice function; all moves, GDA, NSGAII ranking mechanism
Ferreira et al. [53] (2017)	Software Product Line Testing	Using crossover and mutation operators as LLH; HH-based MOEAs (IBEA, SPEA2, NSGAII, and MOEA/D-DRA), namely elitism selection strategy; random and upper confidence
Hitomi and Selva [74] (2017)	MOEAs benchmark (WFG, UF and DTLZ test suite)	HH-based MOEAs (IBEA, MOEA/D-DRA, NSGAII); multiple adaptive operator selections
Xu et al. [75] (2018)	Multiobjective mapping for network-on-chip	Genetic-based hyper-heuristic algorithm; using genetic operator as LLH; reinforcement learning strategy with adaptive weights
Yao et al. [76] (2018)	Multiobjective route planning in a smart city	Reinforcement learning mechanism; domain-LLH;
Almeida et al. [77] (2018)	Permutation flow shop problem	MOEA/D-DRA-based MOHH; MAB; crossover and mutation operators
Gomez and Terashima- Marin [78] (2018)	Bi-objective 2D bin packing problems	Evolutionary hyper-heuristics multiobjective framework; NSGAII, SPEA2 and GDE3
Castro et al. [79] (2018)	MOEAs benchmark (DTLZ, WFG test suite)	Fitness rate rank-based MAB; using leader selection methods and archiving strategies as LLH; R2-based IE acceptance and reward
Zhang et al. [80] (2018)	Software release planning	Extreme value credit assignment; probability matching; domain-LLH
Qian et al. [52] (2018)	MOLCLRP	Tabu search; reinforcement learning method; NSGA-II ranking mechanism
Zhou et al. [81] (2019)	Flexible job shop scheduling	NSGA-II ranking mechanism; Pareto strength; genetic programming
Chand et al. [82] (2019)	resource constrained project scheduling problem	genetic programming hyperheuristic; NSGA-II ranking mechanism; using priority rules as LLH
Li et al. [83] (2019)	MOEAs benchmark	Learning automata; MOEAs as LLHs

**Table 3:** Mean IGD values of 12 approaches for instances.

Set	BiGE	GrEA	IBEA	NSGAII	NSGA-III	NSLS	PEAS-II	SPEA2	QS1	QS2	QS1a	QS2a
L201	2.68	3.41	4.66	2.65	3.05	2.44	2.65	**2.12**	*2.24*	2.43	1.72	***1.64***
L202	1.35	2.09	2.73	1.38	1.55	1.04	1.22	*1.00*	**0.98**	1.23	0.49	***0.47***
L203	1.67	2.64	4.08	1.46	2.14	1.36	1.43	*1.33*	**1.28**	1.39	0.82	***0.75***
L301	2.29	4.68	5.95	2.64	3.15	2.40	2.77	2.23	**1.49**	*1.67*	***0.70***	0.72
L302	3.381	5.01	6.50	3.51	4.34	3.39	3.46	*3.380*	**3.17**	3.90	***2.20***	2.30
L303	4.52	5.87	7.36	5.30	4.53	4.66	4.94	3.796	**3.01**	*3.795*	***2.09***	2.81
L401	*2.37*	3.73	4.36	3.54	3.23	2.90	2.67	2.61	**2.36**	2.87	***1.17***	1.29
L402	*2.59*	3.18	3.48	4.13	3.25	2.99	2.93	2.86	**2.48**	2.84	***1.17***	1.32
L403	*2.57*	4.10	4.93	3.77	3.48	3.01	2.84	2.83	**2.57**	2.98	***1.336***	1.338
L501	**3.61**	5.06	5.89	6.30	4.76	5.60	5.23	4.33	*4.10*	4.43	***1.71***	2.15
L502	3.79	5.74	6.76	5.31	5.19	4.17	5.67	*3.50*	**3.18**	3.95	***1.94***	2.10
L503	*2.99*	3.72	4.19	4.82	3.47	3.85	3.72	3.34	**2.98**	3.68	***1.67***	1.88
f/s/t	1/4/2	0/0/0	0/0/0	0/0/0	0/0/0	0/0/2	0/0/0	1/4/6	10/2/0	0/2/2	9	3

Note: bold numbers indicate the minimum values; italic are the second minimum values; and numbers with underline are the third minimum values; the last two columns are MOHH with archiving methods.

**Table 4:** Mean HV values of 12 approaches for instances.

Set	BiGE	GrEA	IBEA	NSGAII	NSGA-III	NSLS	PEAS-II	SPEA2	QS1	QS2	QS1a	QS2a
L201	2.1467	2.1389	2.1199	2.1422	2.1520	2.1555	2.1401	**2.1680**	*2.1647*	2.1594	2.1699	***2.1769***
L202	0.8204	0.8163	0.8161	0.8184	0.8105	0.8207	0.8180	**0.8221**	*0.8218*	0.8197	0.8251	***0.8252***
L203	**1.7113**	1.7094	1.7007	1.7059	1.7016	1.7091	1.7021	1.7097	*1.7105*	1.7083	1.7151	***1.7154***
L301	1.0995	1.0695	1.0670	1.0732	1.0519	1.0812	1.0555	1.0988	**1.1603**	*1.1511*	***1.1678***	1.1657
L302	*2.8058*	2.7526	2.7432	**2.8109**	2.7704	2.7786	2.7822	2.7596	2.8053	2.7684	***2.8287***	2.8139
L303	1.7902	1.7889	1.7584	1.6971	1.7939	1.7467	1.7393	1.8375	**1.9062**	*1.8478*	***1.9222***	1.8688
L401	*2.0993*	2.0970	2.0870	2.0676	2.0394	2.0973	2.0661	2.0970	**2.1067**	2.0710	***2.1468***	2.1396
L402	2.3967	2.4103	2.4153	2.3796	2.3799	2.4285	2.3836	*2.4312*	**2.4457**	2.3981	***2.4919***	2.4845
L403	2.9053	2.8752	2.8740	2.8989	2.8676	2.9062	2.8898	*2.9091*	**2.9145**	2.8898	***2.9560***	2.9553
L501	2.2779	2.2738	2.1761	2.2312	2.2345	2.2130	2.1681	*2.3257*	**2.3613**	2.3190	***2.4770***	2.4676
L502	2.2264	2.1440	2.1580	2.1760	2.1605	2.2007	2.0440	2.2392	**2.3101**	*2.2517*	***2.3589***	2.3444
L503	2.3465	2.3506	2.3081	2.3510	2.3456	*2.3945*	2.3042	2.3914	**2.4076**	2.3388	***2.4609***	2.4466
f/s/t	1/2/0	0/0/0	0/0/0	1/0/0	0/0/0	0/1/3	0/0/0	2/3/6	8/3/1	0/3/2	9	3

Note: bold numbers indicate the minimum values; italic are the second minimum values; and numbers with underline are the third minimum values; the last two columns are MOHH with archiving methods.

**Table 5:** Scores of each HLH strategy.

	SR	FRR-MAB	QS	CF	Total
AM	110	107	126	115	458
LA	130	116	133	99	478
GDA	0	0	0	0	0
Total	240	223	259	241	NA

**Table 6:** Mean IGD values of 16 pairs.

Set	CRDR	CRD1	CRD2	CRD3	C1DR	C1D1	C1D2	C1D3	C2DR	C2D1	C2D2	C2D3	C3DR	C3D1	C3D2	C3D3
L201	17.6	23.6	16.3	12.5	4.8	9.2	6.5	8.3	3.6	10.6	5.0	3.7	17.0	36.3	24.4	16.2
L202	36.0	41.0	36.1	34.8	4.4	8.5	5.8	9.2	13.3	19.4	16.5	15.4	25.2	29.7	25.5	22.7
L203	22.1	23.1	27.4	21.4	4.5	7.2	6.8	12.6	13.9	13.3	11.2	12.2	19.1	21.5	17.3	16.0
L204	14.4	22.8	17.0	16.6	2.7	6.9	5.9	7.4	6.6	12.4	7.5	10.0	12.6	21.7	15.5	14.0
L301	13.8	19.6	20.6	14.6	4.5	4.2	4.3	8.7	4.8	7.8	5.9	10.0	11.4	32.1	20.5	11.2
L302	17.2	40.5	17.6	17.9	3.2	7.3	4.4	7.2	11.7	14.8	10.1	13.1	27.8	26.9	21.1	16.7
L303	16.7	19.1	16.1	16.3	4.9	6.4	4.8	7.2	7.9	10.9	8.5	9.5	11.0	14.8	10.7	11.0
L304	31.4	36.2	29.4	26.7	4.0	6.6	4.3	11.1	13.1	16.0	11.5	13.0	32.2	36.0	31.4	27.7
L401	9.4	24.3	13.4	10.9	4.5	10.2	4.7	9.2	5.2	14.3	8.8	9.8	7.2	21.5	12.9	6.2
L402	11.6	22.6	13.6	8.8	3.3	6.3	7.1	6.2	6.2	14.1	7.1	7.2	10.3	20.2	9.9	7.1
L403	11.1	23.8	14.0	11.6	3.0	4.3	4.7	10.1	4.1	16.1	8.7	7.6	12.6	25.7	13.4	7.8
L404	9.8	21.2	9.4	12.1	5.1	2.5	7.3	5.5	10.6	10.3	12.1	9.8	11.4	15.0	15.3	10.4
L501	19.7	26.0	19.9	20.0	3.2	1.9	7.3	11.3	10.5	14.4	9.7	10.9	17.4	31.2	19.3	11.8
L502	10.6	29.4	17.4	10.8	2.2	12.8	7.1	7.9	3.1	7.0	7.6	5.8	10.8	28.6	17.8	14.0
L503	14.2	25.8	16.8	13.1	4.5	1.6	3.3	8.6	5.7	17.8	9.4	8.7	13.5	29.3	17.1	13.8
L504	19.2	34.5	22.8	16.9	2.2	2.8	8.0	6.4	11.0	22.8	14.4	11.2	15.2	31.4	22.2	15.0

**Table 7:** Mean HV values of 16 pairs.

Set	CRDR	CRD1	CRD2	CRD3	C1DR	C1D1	C1D2	C1D3	C2DR	C2D1	C2D2	C2D3	C3DR	C3D1	C3D2	C3D3
L201	15.0	12.7	17.6	19.9	25.2	23.0	25.1	23.5	27.4	22.3	25.9	27.4	16.3	6.8	12.4	16.9
L202	9.6	7.0	9.4	10.8	32.8	27.6	30.7	31.9	24.2	19.5	22.6	24.5	15.4	12.1	14.5	17.2
L203	10.9	9.8	7.0	11.4	25.7	24.0	25.7	21.2	14.3	14.6	17.1	16.4	12.8	11.0	13.2	14.6
L204	18.8	12.9	17.2	18.6	32.4	28.5	29.4	29.9	28.4	23.2	27.2	28.3	22.4	16.2	20.5	23.8
L301	19.6	14.9	15.5	19.4	28.3	29.5	28.2	25.7	31.1	27.9	30.5	26.6	23.0	12.1	17.4	23.8
L302	19.1	8.2	17.7	17.6	32.5	29.1	32.2	30.1	22.2	20.9	23.7	21.4	12.6	12.7	16.1	19.7
L303	18.2	16.2	18.1	20.3	30.6	29.6	31.1	32.4	25.2	22.7	25.2	27.2	23.6	20.6	22.9	25.2
L304	0.8	0.5	1.6	2.1	17.1	15.0	16.6	16.5	7.1	5.7	7.0	7.2	0.2	0.0	0.1	0.9
L401	24.8	14.1	21.2	23.7	34.0	25.6	31.4	33.4	29.1	21.5	25.0	30.4	27.9	16.7	22.3	30.1
L402	24.5	17.1	23.0	28.0	35.4	30.3	29.1	32.4	28.4	21.9	27.0	29.1	24.9	17.6	25.7	28.6
L403	22.2	15.0	20.3	22.5	31.9	31.1	32.3	28.4	29.4	19.8	24.3	28.9	22.0	13.6	21.6	26.2
L404	33.0	22.9	32.1	33.9	40.0	40.1	35.6	42.3	31.8	30.7	29.0	34.6	32.3	27.8	27.0	33.1
L501	16.6	12.4	15.7	16.5	29.5	29.0	23.1	22.9	20.8	17.7	20.9	22.8	16.1	9.2	15.7	20.9
L502	18.9	9.6	14.4	18.3	27.9	19.8	22.6	23.7	25.3	22.4	22.1	23.4	18.4	10.3	14.9	16.5
L503	20.8	14.2	19.2	22.1	30.0	31.8	32.0	30.7	28.6	19.4	25.2	27.1	21.6	12.6	19.4	22.0
L504	22.2	12.9	19.1	24.1	37.8	34.5	30.8	32.7	27.7	19.2	24.1	28.1	25.1	14.4	19.8	25.4

**Table 8:** Mean IGD, HV, and RNI values of each fleet composition.

Instance	IGD				HV				RNI			
	L1	L2	M	HF	L1	L2	M	HF	L1	L2	M	HF
L201	36.70	4.27	1.72	0	0.04	18.46	21.94	22.62	0	13.45	8.07	100
L202	15.06	0.94	3.83	0	2.43	8.10	7.57	8.26	0	12.40	1.24	100
L203	12.01	0.83	2.99	0	10.18	17.78	16.88	17.94	0	36.01	5.68	100
L301	12.64	0.81	3.37	0	6.15	12.85	11.87	12.96	0	33.06	5.78	100
L302	28.18	2.37	1.59	0	10.45	28.80	29.64	30.80	0	26.80	21.97	100
L303	26.37	1.76	2.67	0	3.20	19.50	18.91	20.20	0	2.62	0.52	100
L401	24.90	3.17	1.51	0	16.68	31.34	31.90	35.03	0.51	4.33	10.90	100
L402	33.68	4.59	1.13	0	4.85	20.63	24.70	25.70	0	8.11	4.28	100
L403	32.74	3.34	1.26	0	9.33	27.24	29.20	29.96	0	18.18	31.37	100
L501	27.56	3.44	1.25	0	16.03	31.90	36.56	37.74	0.02	3.06	3.78	100
L502	47.84	9.33	1.08	0	0	18.74	26.09	26.60	0	1.41	15.71	100
L503	26.50	4.47	2.77	0	14.21	28.66	30.08	32.06	4.96	4.13	1.45	100

**Table 9:** Mean IGD, HV, and RNI values of each area ratio.

Set	IGD					HV					RNI				
	EQ	Do	TF	FF	ORI	EQ	Do	TF	FF	ORI	EQ	Do	TF	FF	ORI
L201	13.53	6.23	3.14	0.36	3.23	6.64	11.54	13.51	15.24	13.44	0	0	4.17	86.67	9.17
L202	8.77	8.49	1.12	0.52	1.83	8.42	8.62	11.32	11.74	11.07	0.52	2.61	15.65	61.91	19.30
L203	6.44	4.59	3.58	0.08	4.25	20.36	22.61	23.84	26.38	22.94	1.18	2.19	0	94.95	1.68
L301	11.52	3.35	1.96	0.32	1.64	9.69	12.72	13.25	15.09	13.29	0	0	12.80	61.20	26.01
L302	7.29	1.84	1.07	0.66	0.98	22.05	26.92	27.65	28.02	27.45	0.59	10.42	11.68	48.85	28.46
L303	18.99	4.51	3.65	0.14	3.57	10.24	19.43	20.11	23.43	20.21	0	0	3.16	92.21	4.62
L401	7.70	2.63	1.11	0.59	1.33	16.11	20.24	20.77	20.85	22.04	0	0.66	27.21	40.48	31.65
L402	17.83	7.00	2.35	0.03	5.82	12.68	18.15	21.75	23.75	19.50	0	0	2.12	97.88	0
L403	10.29	5.16	3.66	0.11	3.46	19.90	22.92	24.41	28.88	24.58	0	0	0.20	97.49	2.31
L501	10.54	7.25	1.41	1.29	0.31	15.72	19.55	24.24	24.42	26.73	0	0	4.90	22.67	72.43
L502	6.79	4.00	0.95	0.83	0.86	19.61	21.33	23.87	23.84	24.21	0.06	0.63	19.19	48.45	31.67
L503	13.37	6.41	1.34	0.55	1.05	15.60	21.33	25.31	25.96	25.37	0	0	12.28	58.93	28.79
